# Nonlinear association between PD-L1 expression levels and the risk of postoperative recurrence in non-small cell lung cancer

**DOI:** 10.1038/s41598-024-66463-6

**Published:** 2024-07-04

**Authors:** Kensuke Kojima, Hironobu Samejima, Takafumi Iguchi, Toshiteru Tokunaga, Kyoichi Okishio, Hyungeun Yoon

**Affiliations:** 1 Department of General Thoracic Surgery, NHO Kinki Chuo Chest Medical Center, 1180 Nagasone-Cho, Kita-ku, Sakai-shi, Osaka Japan; 2 Clinical Research Center, NHO Kinki Chuo Chest Medical Center, Osaka, Japan; 3 Department of Thoracic Oncology, NHO Kinki Chuo Chest Medical Center, Osaka, Japan

**Keywords:** Machine learning, Random forest, Multivariate Cox proportional hazard model, Non-small cell lung cancer, Recurrence-free survival, Lung cancer, Medical research, Risk factors

## Abstract

Accurate prediction of postoperative recurrence is important for optimizing the treatment strategies for non-small cell lung cancer (NSCLC). Previous studies identified the PD-L1 expression in NSCLC as a risk factor for postoperative recurrence. This study aimed to examine the contribution of PD-L1 expression to predicting postoperative recurrence using machine learning. The clinical data of 647 patients with NSCLC who underwent surgical resection were collected and stratified into training (80%), validation (10%), and testing (10%) datasets. Machine learning models were trained on the training data using clinical parameters including PD-L1 expression. The top-performing model was assessed on the test data using the SHAP analysis and partial dependence plots to quantify the contribution of the PD-L1 expression. Multivariate Cox proportional hazards model was used to validate the association between PD-L1 expression and postoperative recurrence. The random forest model demonstrated the highest predictive performance with the SHAP analysis, highlighting PD-L1 expression as an important feature, and the multivariate Cox analysis indicated a significant increase in the risk of postoperative recurrence with each increment in PD-L1 expression. These findings suggest that variations in PD-L1 expression may provide valuable information for clinical decision-making regarding lung cancer treatment strategies.

## Introduction

According to the latest projections from the American Cancer Society, 2,001,140 new cancer cases and 611,720 cancer deaths are expected to occur in the United States by 2024, underscoring the persistent burden of this complex group of diseases despite recent advances in prevention, early detection, and treatment^1^. Among these, non-small cell lung cancer (NSCLC) remains a highly lethal malignancy worldwide^2^. Surgical resection is an effective therapeutic approach for treating NSCLC. Approximately 15–38% of patients who undergo NSCLC resection experience recurrence^3^. Predicting and preventing postoperative recurrence remains major clinical challenges. Therefore, there is an urgent need to clinically assess the risk of postoperative NSCLC recurrence and identify novel predictive factors.

PD-L1 (programmed death ligand 1) and PD-1 (programmed death 1) are factors associated with the immune evasion mechanism of tumors^4^. PD-L1 expressed on tumor cells interacts with PD-1 expressed on immune cells, suppressing the activity of immune cells and promoting immune evasion by tumor cells^5^. Immune checkpoint inhibitors (ICIs) disrupt this immune evasion mechanism and exert immunological anti-tumor effects^6,7^. Clinical studies have reported that higher PD-L1 expression levels in NSCLC are correlated with increased efficacy of immune checkpoint inhibitors against tumors^8,9^. Paradoxically, this suggests that increased expression of PD-L1 may lead to greater immune suppression, potentially increasing the risk of postoperative recurrence in NSCLC. Although this hypothesis is supported by several reports, including our previous studies^10,11^, conflicting reports suggest that the expression of PD-L1 is a favorable prognostic factor in NSCLC^12,13^. Thus, a consensus on the role of PD-L1 in the risk of postoperative recurrence of NSCLC remains elusive, and further discussion is warranted.

To address this gap, we conducted a study to elucidate the association between the risk of postoperative recurrence and PD-L1 expression in NSCLC patients. Using machine learning, we constructed a postoperative recurrence prediction model based on the clinical and pathological features of patients who had undergone NSCLC resection. By analyzing this prediction model, we evaluated the contribution of differences in the expression of PD-L1 to postoperative recurrence and explored the relationship between increased PD-L1 expression and increased recurrence risk.

The significance of our study is that it provides novel insights into the prediction of postoperative recurrence based on the expression of PD-L1 in patients with NSCLC. Unexplored insights from a machine learning approach may help improve the accuracy of the prediction of postoperative recurrence and may be useful for developing lung cancer treatment strategies tailored to PD-L1 expression levels. The results of our study could potentially provide new guidelines for recurrence prevention strategies in patients undergoing lung cancer resections.

## Materials and methods

### Patients

The study population consisted of 647 patients who underwent lung cancer resection at the NHO Kinki Chuo Chest Medical Center (KCMC) between April 2017 and June 2022. Only patients with a histologically confirmed pathologically complete resection (R0) were included. Patients with incomplete tumor removal (R1) were excluded from the study. Patient background information was retrospectively collected from electronic medical records. Patients for whom electronic medical records were unavailable were also excluded. Histopathological diagnoses were performed by institutional pathologists in accordance with the 2015 World Health Organization classification^14^. Eligible patients who provided informed consent were treated with platinum-based adjuvant chemotherapy, based on the guidelines of the Japanese Lung Cancer Association. Clinicopathological features, including age, sex, neutrophil-to-lymphocyte ratio (NLR) before surgery, pathological stage (American Joint Committee eighth edition), histological type, vascular invasion (v), lymph vessel invasion (Ly), adjuvant chemotherapy, PD-L1 expression, and postoperative recurrence-free survival (RFS), were collected from medical records as key features for the machine learning model and statistical analyses. Pathological information (i.e., pathological stage, v, Ly, and histological type) was collected based on the pathology reports created by experienced pathologists. To focus on the impact of the expression of PD-L1 on the risk of postoperative recurrence in patients undergoing surgical resection as the primary treatment modality, we applied the following exclusion criteria: (1) patients who received perioperative immunotherapy, as this treatment modality was not yet approved in Japan during the study period; (2) patients who underwent induction therapy prior to surgical resection; and (3) patients who received preoperative radiotherapy. These exclusion criteria were based on previous reports suggesting that induction therapy and preoperative radiotherapy can influence PD-L1 expression levels in patients with NSCLC, which would potentially confound the relationship between the baseline PD-L1 expression and postoperative outcomes ^15,16^. Patients who received treatments that may alter the expression of PD-L1 before surgery were excluded with the aim of evaluating the relationship between the baseline expression of PD-1 and the risk of postoperative recurrence in NSCLC patients, while accounting for the potential impact of adjuvant treatments on outcomes.

This study was approved by the KCMC Institutional Ethics Committee (Approval No. 2023–31), which granted a retrospective exemption from obtaining informed consent from all study participants, considering the anonymous nature of the study. The KCMC website offers information on opting for participation in the study. All research methods adhered to the applicable guidelines and regulations.

### The RFS

The primary endpoint of this study was the assessment of postoperative recurrence based on the clinical diagnosis. Recurrence-free survival (RFS) was defined as the period between lung cancer resection and the clinical confirmation of recurrence. We considered patients to be recurrence-free until definitively confirmed after surgery. Patients who underwent surgery underwent regular blood sampling and radiographic examinations every three–six months. In cases where abnormal findings suggestive of disease recurrence were observed, additional diagnostic tests, such as magnetic resonance imaging (MRI) of the head, contrast-enhanced computed tomography (CT), positron emission tomography (PET), and pathological examination of tissue biopsy samples were conducted. Recurrence was diagnosed through a comprehensive evaluation of the test results during joint conferences involving general thoracic surgeons, oncologists, pathologists, and radiologists.

### PD-L1 immunohistochemistry

A pathologist examined all cancer cells detectable in the tissue samples extracted from the resected lung cancer specimens. The PD-L1 clone 22C3 pharmDx kit was used in conjunction with the Dako Automated Link 48 platform (Agilent Technologies, Santa Clara, CA, USA) for immunohistochemical analysis to assess the expression of programmed death-ligand 1 (PD-L1). The tumor proportion score (TPS) for PD-L1 was calculated as the percentage of membranous staining observed in the tissue samples either complete or partial. The score ranged from 0 to 100% and was calculated using the standard 22C3 assay protocol. The tumor region was visually segmented into four areas, and the proportion of PD-L1 positive cells in each area was quantified, resulting in an average value for the clinical TPS.

### Machine learning

We used a machine learning approach to develop a postoperative recurrence prediction model for lung cancer. We chose machine learning algorithms with high predictive accuracy, including bagging and boosting techniques from the ensemble learning family, as well as random forest, gradient boosting, light-gradient boosting, and Ada boosting^17,18^. We selected variables for the machine learning algorithm based on factors previously reported to correlate with postoperative recurrence, in addition to the expression of PD-L1. The following variables were selected: the expression of PD-L1 (TPS), pathological stage (stages I–III), invasion of blood vessels (v0–v1)^19^, lymphatic vessel invasion (Ly0–Ly1)^20^, histological classification of cancer (adenocarcinoma, squamous cell carcinoma, or others), neutrophil-to-lymphocyte ratio (NLR)^21^, adjuvant chemotherapy^22^, age, and sex. We assessed the correlations of individual variables within the machine learning model through heat map analysis. To prevent overfitting, the entire dataset was randomly divided into three parts: training, validation, and test sets. The training set comprised 80% of the dataset, wheresa the validation and test sets comprised 10% each.

Python packages (RandomForestClassifier, lgb.LGBMClassifier, GradientBoostingClassifier, and AdaBoostClassifier) were used to construct the machine-learning models. Training data were employed to train the model and explore hyperparameters. By employing optimal hyperparameters, the performance of the model was assessed using the validation data. Finally, a conclusive evaluation of the model was conducted using the test data. We employed Bayesian hyperparameter optimization using the Python library Optuna, to select the hyperparameters for each machine learning model designed to predict postoperative recurrence. For each model, we conducted 100 trials to determine the optimal hyperparameter configuration. Subsequently, we selected the hyperparameters linked to the most effective configuration as the optimal settings for each investigated model (Table S1). The predictive performance of each model was evaluated quantitatively using metrics such as the accuracy, F1 score, Brier score, receiver operating characteristic area under the curve (ROC AUC), and precision-recall AUC (PR AUC). The model with the best predictive performance is used as the final model. The predictions were interpreted using the Shapley additive explanation (SHAP) values^23^. SHAP values are derived from Shapley values in coalition game theory, offering a robust and precise approach for quantifying the impact of individual variables on the predictions of a machine learning model. SHAP values were computed using the shap v0.28.5 Python library and visualizations were generated using Matplotlib v3.0.311. To investigate the influence of PD-L1 expression on the model predictions, we utilized the plot_partial_dependence function from the scikit-learn Python package and visualized the influence using partial dependence plots.

### Statistical analysis

To evaluate the contribution of PD-L1 expression to postoperative recurrence, we used a multivariate Cox proportional hazard model. In this analysis, the outcome variable was postoperative recurrence, and the explanatory variable of interest was PD-L1 expression in the resected lung cancer tissues. PD-L1 expression was treated as a continuous variable, independent of the threshold. Features, other than the PD-L1 expression used in the machine learning model were incorporated as confounders. Notably, in a multivariate Cox proportional hazards model, the number of variables that can be included is limited by the number of outcome events. In our study, 151 recurrence cases (151), allowed for up to 15 variables to be included in the model^24^. The primary variable in our study was the expression of PD-L1. Ten other confounding variables were selected from our model. Specifically, we considered age (categorized as ≥ 65 and < 65 years, with < 65 years as the reference group), sex (male and female, with male as the reference), pathological stage (categorized as stages I–III, with stage I as the reference), histological type (categorized as adenocarcinoma, squamous cell carcinoma and others, with adenocarcinoma as the reference), invasion of lymphatic vessels (categorized as Ly0–Ly1, with Ly0 as the reference), invasion of blood vessels (categorized as v0–v1, with v0 as the reference), NLR (continuous variable), and adjuvant chemotherapy. We assessed the validity of the proportional hazard assumption in the Cox models by examining the martingale residual plots and the Schoenfeld residual test. Multicollinearity among the variables in the multivariable Cox proportional hazards model was evaluated using a variance inflation factor (VIF) with a threshold of < 2 to determine its presence or absence.

Statistical analyses were performed using Easy R (EZR) (Saitama Medical Center, Saitama, Japan), which is a graphical user interface of R (The R Foundation for Statistical Computing, Vienna, Austria)^25^. EZR is an improved version of R commander with additional biostatistical functions. Statistical significance was set at P < 0.05.

### Ethical approval

This study was approved by the Institutional Review Board (IRB) of the National Hospital Organization Kinki　Chuo Chest Medical Center (KCMC) (approval number: 2023–31) and was carried out in accordance with the Declaration of Helsinki. The IRB of KCMC waived the requirement for informed consent from all research participants because of the retrospective and anonymous nature of the study. Information about opting out of this study is provided on the KCMC homepage.

### Patient consent statement

Owing to the retrospective nature of this study, informed consent from the patients was not required.

## Results

### Patient characteristics

**Figure 1:**
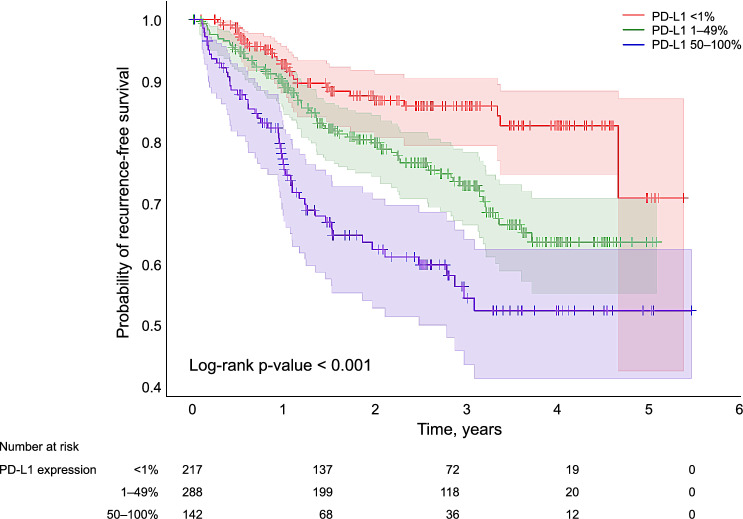
Kaplan–Meier curve showing the probability of recurrence-free survival among patients after lung cancer resection according to the PD-L1 expression levels.

The cohort consisted of 647 patients who were randomly stratified into the training (517 patients, 80%), validation (65 patients, 10%) and test groups (65 patients, 10%). The incidence rate of postoperative recurrence was 23% and 151 patients were observed. The median PD-L1 expression level in resected lung cancer tissue for the entire cohort was 5% (range, 0–39%). In the cohort, 73% were ≥ 65 years of age and 59% were male. The pathological subtypes of lung cancer identified in our study were adenocarcinoma (74%, 478 cases), squamous cell carcinoma (18%, 116 cases), and other subtypes (8%, 53 cases). The pathological stages were as follows: stage I, 71%; stage II, 17%; and stage III, 12%. The median RFS was 729 days (range, 360–1157 days). No statistically significant differences were observed among the three subgroups for any of the variables, indicating homogeneous distribution (Table 1). In the total cohort, patients were classified into three groups based on tPD-L1 expression levels: no expression (< 1%), low expression (1–49%), and high expression (50–100%). The probability of postoperative RFS in these three groups was compared using Kaplan–Meier curves. The analysis revealed that RFS was significantly shorter in the high-, low-, and no-expression groups (Fig. 1).

**Table 1:** Clinical characteristics and outcomes of 647 patients undergoing lung cancer resection.

Characteristic	Total cohort (n = 647)	Training cohort (n = 517)	Validation cohort (n = 65)	Test cohort (n = 65)	P value
Continuous variables, median (Q1, Q3)					
PD-L1 expression (TPS [%])	5 (0–39)	5 (0–40)	5 (0–40)	3 (0–20)	0.83
NLR	1.8 (1.4–2.4)	1.8 (1.4–2.5)	1.8 (1.4–2.2)	1.9 (1.4–2.5)	0.79
Number of recurrent cases	151 (23)	129 (25)	10 (15)	12 (19)	0.14
RFS (day)	729 (360–1157)	730 (363–1172)	736 (336–1292)	647 (368–1105)	0.86
Categorical variables, n (%)					
Age (≥ 65)	472 (73)	380 (74)	50 (77)	42 (65)	0.24
Male sex	381 (59)	301 (58)	39 (60)	41 (63)	0.74
Histological types					0.35
Adenocarcinoma	478 (74)	383 (74)	43 (66)	52 (80)	
Squamous cell carcinoma	116 (18)	90 (17)	17 (26)	9 (14)	
Others^†^	53 (8)	44 (9)	5 (8)	4 (6)	
Pathological stage					0.98
Stage I	456 (71)	364 (70)	45 (69)	47 (72)	
Stage II	112 (17)	90 (17)	11 (17)	11 (17)	
Stage III	79 (12)	63 (12)	9 (14)	7 (11)	
Vascular invasion					0.89
v0	200 (31)	162 (31)	19 (29)	19 (29)	
v1	447 (69)	355 (69)	46 (71)	46 (71)	
Lymph vessel invasion					0.35
Ly0	246 (38)	193 (37)	23 (35)	30 (46)	
Ly1	401 (62)	324 (63)	42 (65)	35 (54)	
Adjuvant chemotherapy	66 (10)	48 (9)	11 (17)	7 (11)	0.16

^†^Histological types excluding adenocarcinoma and squamous cell carcinoma in non-small cell lung cancer were as follows: among 53 cases, 17 had pleomorphic carcinoma, 16 had large-cell neuroendocrine carcinoma, 12 had adenosquamous carcinoma, 5 had large-cell carcinoma, and 3 had carcinoid tumors.

*NLR* neutrophil-to-lymphocyte ratio, *PD-L1* programmed death-ligand 1, *RFS* recurrence-free survival, *TPS* tumor proportion score.

### Evaluation of machine learning models

**Figure 2:**
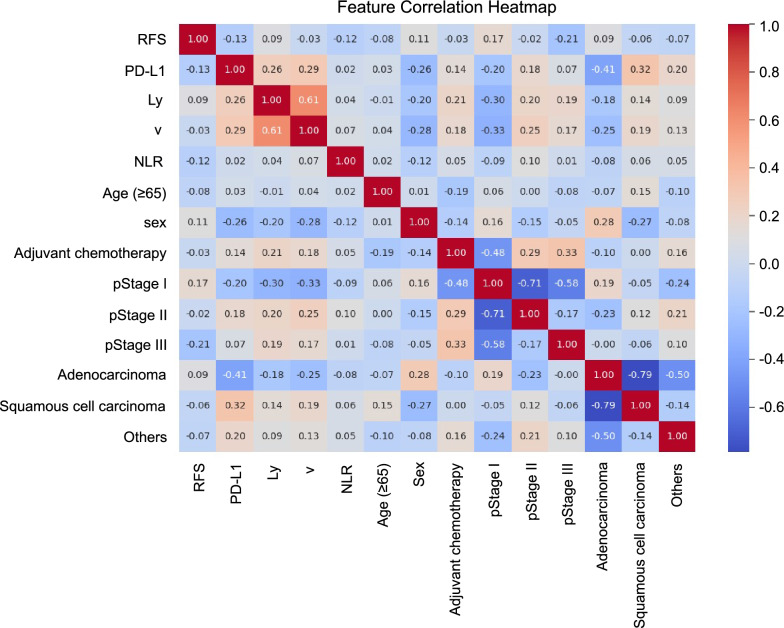
Heatmap showing the correlation coefficients between different features in a dataset. Colors range from positive correlation (red) to negative correlation (blue).

The correlation between the features to be input into the machine learning model was examined using a heat map (Fig. 2). The correlation coefficients between features from different categories were all < 0.7, suggesting that there was no strong correlation. Machine learning models, including random forest, gradient boosting, light gradient boosting, and Ada boosting algorithms, were trained using the training dataset for postoperative recurrence prediction. Table S2 shows the performance of the machine learning models using the training dataset. The model performance was assessed by measuring the ROC AUC, PR AUC, accuracy, F1 score, and Brier score. In the training dataset, the random forest model demonstrated the highest overall performance, with an ROC AUC of 0.99 (95% CI 0.99–1.00), PR AUC of 1.00 (95% CI 1.00–1.00), accuracy of 0.99 (95% CI 0.97–1.00), F1 score of 0.98 (95% CI 0.96–0.99) and Brier score of 0.04 (95% CI 0.02–0.06). The performance of each model was then assessed by using a validation dataset. Among all prediction models, the random forest model showed the highest performance with an ROC AUC of 0.97 (95% CI 0.90–1.00), PR AUC of 0.86 (95% CI 0.72–1.00), Accuracy of 0.92 (95% CI 0.81–1.00), F1 score of 0.76 (95% CI 0.58–0.94) and Brier score of 0.08 (95% CI 0.01–0.16) (Table 2). Based on the overall performance, the random forest model was selected as the superior model. Finally, the performance of the random forest model was assessed on the test data and showed the following predictive accuracies: ROC AUC of 0.90 (95% CI 0.78–1.00), PR AUC of 0.78 (95% CI 0.61–0.94), accuracy of 0.92 (95% CI 0.81–1.00), F1 score of 0.74 (95% CI 0.55–0.92) and Brier score of 0.10 (95% CI 0.02–0.18) (Table 3).

**Table 2:** Predictive performance of various machine learning models in identifying postoperative lung cancer recurrence in the validation cohort.

Machine learning model	Assessment metrics (95% CI)				
	ROC AUC	PR AUC	Accuracy	F1 score	Brier score
Random forest	0.97 (0.90–1.00)	0.86 (0.72–1.00)	0.92 (0.81–1.00)	0.76 (0.58–0.94)	0.08 (0.01–0.16)
Gradient boosting	0.92 (0.81–1.00)	0.73 (0.54–0.92)	0.89 (0.76–1.00)	0.67 (0.47–0.86)	0.08 (0.01–0.14)
Light gradient boosting	0.93 (0.82–1.00)	0.62 (0.42–0.82)	0.89 (0.76–1.00)	0.67 (0.47–0.86)	0.07 (0.01–0.15)
Ada boosting	0.93 (0.82–0.91)	0.61 (0.41–0.81)	0.89 (0.76–1.00)	0.64 (0.44–0.83)	0.21 (0.08–0.34)

*CI* confidence interval, *PR AUC* area under the precision-recall curve, *ROC AUC* area under the receiver operating characteristic curve.

**Table 3:** Predictive performance of a random forest model in identifying postoperative lung cancer recurrence in the test cohort.

Machine learning model	Assessment metrics (95% CI)				
	ROC AUC	PR AUC	Accuracy	F1 score	Brier score
Random forest	0.90 (0.78–1.00)	0.78 (0.61–0.94)	0.92 (0.81–1.00)	0.74 (0.55–0.92)	0.10 (0.02–0.18)

*CI* confidence interval, *PR AUC* area under the precision-recall curve, *ROC AUC* area under the receiver operating characteristic curve.

### Importance and dependence of features in machine learning predictions

**Figure 3:**
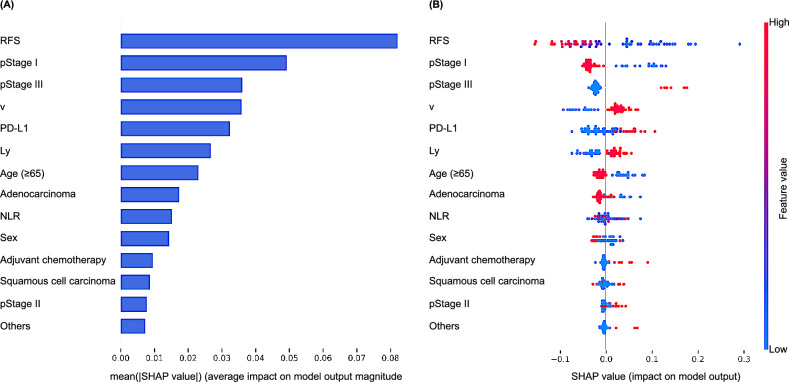
(**A**) Variables with the most significant impact on the prediction of postoperative recurrence ranked in order of importance. (**B**) The distribution of the influence of each variable on the prediction of postoperative recurrence is presented. The numerical characteristics of the variables are visually represented by colors, with larger values shown in red and smaller values shown in blue. Negative SHAP values (spread to the left) suggest a decrease in the probability of postoperative recurrence, while positive values (spread to the right) suggest an increase in the probability.

**Figure 4:**
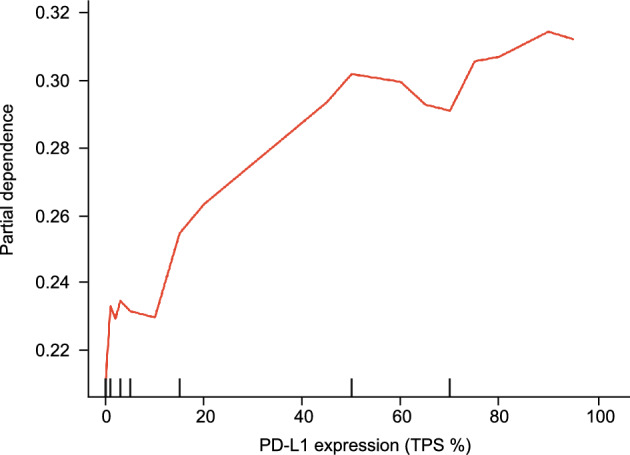
A partial dependence plot depicting the association between the PD-L1 expression levels and the prediction of postoperative recurrence. The x-axis represents the PD-L1 expression levels, while the y-axis represents the contribution to the prediction of postoperative recurrence (partial dependency).

To assess the importance of each feature used in the prediction model, a SHAP analysis was performed using a random forest model. Among the features analyzed, the average SHAP values were the highest for RFS, pathological stage I, pathological stage III, and vascular invasion, followed by PD-L1 expression (Fig. 3A). The distribution of the SHAP scores was also analyzed for each feature (Fig. 3B). With respect to the PD-L1 expression, the red dots were predominantly located in the positive SHAP value region, whereas the blue dots were located in the negative SHAP value region. Thus, an increase in the expression of PD-L1 was associated with an increase in its contribution to the model predictions. To further explore the impact of PD-L1 expression on model predictions, partial dependence plots were generated (Fig. 4). The dependence on predictions greatly increased when PD-L1 was expressed in compared to when it was not, and a subsequent linear increase in dependency was observed with increasing PD-L1 expression levels.

### Multivariate Cox proportional hazards analysis

We then examined the association between PD-L1 expression and postoperative recurrence using a statistical approach, specifically a multivariate Cox proportional hazards model. The results of the multivariate Cox proportional hazards analysis are presented in Table 4. Multicollinearity of each explanatory variable was assessed using the variance inflation factor (VIF). The VIF was less than 2 for all variables, indicating the absence of multicollinearity (Table S3). A proportional hazard analysis was performed using a Martingale residual plot. The general horizontal trend of this curve confirmed the fulfilment of the proportional hazards assumption (Figure S1). In addition, the proportional hazards assumption was evaluated for all explanatory variables included in the Cox models for RFS using the Schoenfeld residual test (Table S4). The P-values for each variable were greater than 0.05, indicating that the proportional hazards assumption was satisfied. An increase in the expression of PD-L1 was associated with a significantly increased risk of postoperative recurrence (adjusted hazard ratio, 1.006; 95% CI, 1.000–1.011).

**Table 4:** Results of a multivariate Cox proportional hazard analysis of RFS according to the expression of PD-L1.

	Unadjusted HR (95% CI), P	^†^Adjusted HR (95% CI), P
PD-L1 expression (TPS [%])	1.013 (1.008–1.018), < 0.001	1.006 (1.000–1.011), 0.04

^†^In the multivariate analysis, the hazard ratio was adjusted for age, sex, histological type, pathological stage, vascular invasion, lymph vessel invasion, and adjuvant chemotherapy.

*CI* confidence interval, *HR* hazard ratio, *PD-L1* programmed death ligand 1, *RFS* recurrence-free survival.

## Discussion

We conducted a single-center retrospective observational study of 647 patients with postoperative NSCLC to investigate the association between the expression of PD-L1 and postoperative recurrence. In our cohort, the recurrence rate was 23.3%, consistent with previously reported rates of 20–26% in larger cohorts studying lung cancer^26,27^. Our study showed that groups with higher PD-L1 expression levels had shorter RFS in the conventional classification based on PD-L1 expression levels (no expression [< 1%], low expression [1–49%], and high expression [50–100%]). In addition, using a machine learning model with a random forest algorithm and a multivariate Cox proportional hazards model with statistical analysis, we investigated the impact of PD-L1 expression on the postoperative recurrence of NSCLC. Our results showed a nonlinear increase in the risk of postoperative recurrence based on PD-L1 expression level.

Including our previous study and several conventional studies, reports have linked PD-L1 expression in NSCLC to an increased risk of postoperative recurrence^11,28–31^. However, the results of our study may help further develop this association. In previous studies, a statistical approach has demonstrated an association between the expression of PD-L1 in NSCLC and postoperative recurrence. This association was also observed in the multivariate Cox proportional hazards analysis in our study, which is consistent with previous research. In contrast to previous studies, our study statistically demonstrated an increase in postoperative recurrence risk corresponding to each 1% increase in PD-L1 expression level by conducting statistical analysis treating PD-L1 expression as a continuous variable rather than as a categorical variable. Furthermore, we used a machine learning approach to evaluate the effect of PD-L1 expression on postoperative recurrence in detail. Specifically, we revealed in detail the nonlinear relationship between postoperative recurrence and PD-L1 expression level using SHAP analysis and partial dependence plots. Our machine learning approach has discovered a new finding that has not been reported in previous statistical analyses. This finding highlights the potential for a nonlinear relationship between PD-L1 expression levels and postoperative recurrence of NSCLC. In conventional studies, the relationship between the expression of PD-L1 and postoperative prognosis has been examined using categorical classifications, such as no expression (< 1%), low expression (1–49%), and high expression (≥ 50%). However, our study leveraging machine learning techniques revealed a more nuanced, continuous association between PD-L1 expression levels and recurrence risk that could not be captured by these traditional categories. The nonlinear increase in the risk of recurrence with even minimal PD-L1 levels (as low as 1%) and the linear escalation of risk when the expression increased beyond 1% suggests the importance of considering PD-L1 expression as a continuous variable rather than a discrete category. This finding underscores the potential for variations in postoperative recurrence risk across the spectrum of PD-L1 expression, providing a more granular understanding of the relationship between the expression of PD-L1 and NSCLC recurrence. The results of this study may contribute to its clinical applications. Adjuvant chemotherapy with immune checkpoint inhibitors during the perioperative period of lung cancer improves a patient’s prognosis, even with PD-L1 expression levels as low as 1%, and this effect becomes more pronounced when the expression levels exceed 50%^32^. The nonlinear increase in the risk of recurrence based on PD-L1 expression levels may encourage the active introduction of perioperative immune checkpoint inhibitors in patients with PD-L1-positive lung cancer as a clinical decision-making strategy.

The results of this study can be explained from an immunological perspective as follows. We showed that the contribution to recurrence increased non-linearly and sharply when PD-L1 was expressed, even at 1%, comparied to PD-L1-negative cases. This trend suggests that even minimal PD-L1 expression may have a significant impact on postoperative recurrence in NSCLC. Even when 1% of cancer cells express PD-L1, subtle interactions between PD-L1-expressing cancer cells and the surrounding immune cells may lead to local immune suppression. This local suppression of immune cell activity may increase the immune resistance of cancer cells, activate local immune escape mechanisms, and potentially increase the risk of postoperative recurrence. This possibility is supported by clinical studies showing higher efficacy of immune checkpoint inhibitors in cases where PD-L1 is expressed, even at 1%, in comparison to cases with no expression^33,34^. In contrast, in cases with PD-L1 expression levels ≥ 1%, a linear increase in the contribution to postoperative recurrence was observed with increasing expression levels. This mechanism suggests that as the number of cancer cells expressing PD-L1 increases, the suppression of immune cell activity increases, resulting in more immune-resistant cancer cells and further activation of immune escape mechanisms. This possibility is supported by clinical trials that demonstrated the higher efficacy of immune checkpoint inhibitors in patients with high PD-L1 expression levels (≥ 50%) compared to those with low expression levels (1–49%) and PD-L1-negative cases (< 1%)^34–36^. In addition to direct interactions with immune cells, the involvement of PD-L1 in the formation of the tumor microenvironment could be a potential mechanism by which different PD-L1 expression levels exert a nonlinear influence on the risk of recurrence. PD-L1 is capable of nuclear translocation and has been reported to directly bind to DNA and regulate the transcriptional induction of genes involved in the tumor microenvironment, such as immune responses and inflammation. In other words, when PD-L1 is expressed, the higher its expression level, the more likely it is that signaling pathways associated with tumor immune evasion are activated, potentially contributing to the establishment of an immunosuppressive tumor microenvironment. Formation of the tumor microenvironment may be closely related to the survival and proliferation of residual cancer cells, possibly increasing the risk of postoperative recurrence^37^. The nonlinear changes in the increased risk of recurrence as a function of PD-L1 expression levels revealed in our study suggest significant variations in the risk of recurrence, even within the PD-L1 expression categories of low (1–49%) and high (50–100%) that are commonly used in clinical practice. Our study is the first to reveal the machine learning-based nonlinear variation of postoperative recurrence risk dependent on the continuous range of PD-L1 expression levels from low to high expression.

This study has several limitations. First, as this was a retrospective observational study conducted at a single institution, caution should be exercised when generalizing the results. Given the retrospective nature of the study, it is possible that clinical factors other than the expression of PD-L1 were not considered. Further validation through additional studies, such as prospective investigations or multicenter collaborations, is essential to elucidate the impact of these factors. Second, the performance of machine learning models is strictly limited by their predictive accuracy and requires careful consideration in clinical applications. The results of this study, derived from a limited dataset, require further validation in clinical research using other cohorts or larger datasets to determine whether similar trends can be observed in recurrence-prediction models. Third, the random survival forest algorithm is known to be more appropriate for a survival time analysis than the random forest. algorithm However, we chose the random forest model because the primary objective of our study was to perform a detailed analysis of the impact of the expression of PD-L1 on the prediction of postoperative recurrence. Using the random forest model, we were able to apply a SHAP analysis and partial dependence plot analysis to reveal the nonlinear influence of the expression of PD-L1 on the prediction of postoperative recurrence. At present, there are no packages available in Python or R that can directly perform these analyses on the random survival forest model, making it the best choice for our study. However, it is important to note that the random forest model may not be as robust as the random survival forest model for a survival time analysis. To overcome this limitation, we employed a multivariate Cox proportional hazards model to statistically validate the contribution of the expression of PD-L1 to postoperative recurrence. In future studies, when the implementation of SHAP and PDP analyses becomes available for the random survival forest model, it would be desirable to use these methods to further analyze the relationship between the expression of PD-L1 and postoperative recurrence in greater detail. Overall, these considerations highlight the need for cautious interpretation and future research to improve the robustness and applicability of the findings.

## Conclusion

In conclusion, our study using machine learning and statistical analysis revealed a significant nonlinear association between the expression of PD-L1 and risk of postoperative recurrence in NSCLC. We demonstrated that even minimal PD-L1 expression levels (as low as 1%) are associated with an increased risk of recurrence, suggesting the potential impact of subtle immune interactions. Furthermore, a continuous increase in the expression of PD-L1 beyond 1% corresponded to a linear increase in the risk of recurrence. These novel findings suggest a nonlinear relationship between the expression of PD-L1 and postoperative recurrence, and provide valuable insights into personalized therapeutic strategies for NSCLC. Our findings highlight the importance of considering the continuum of PD-L1 expression levels in real-world clinical practice, as opposed to the traditional classification of PD-L1 expression levels as low or high. This study contributes to a better understanding of the immunological factors influencing NSCLC recurrence, paving the way for tailored treatment interventions based on the expression of PD-L1.

### Supplementary Information

Supplementary Information 1.

Supplementary Information 2.

Supplementary Information 3.

Supplementary Information 4.

Supplementary Information 5.

## Data Availability

The database used in this study is not available to the public. Participants in our study did not agree that their data were publicly shared. The Python code used in this study is available upon request from the corresponding author, k7kensuke@icloud.com.
