# Supplementary figures and images for: Nonlinear association between PD-L1 expression levels and the risk of postoperative recurrence in non-small cell lung cancer
Source: Sci Rep. 2024 Jul 4;14:15369. doi: 10.1038/s41598-024-66463-6 (PMC11224325; doi:10.1038/s41598-024-66463-6)

**Figure S1. The evaluation of the proportional hazards assumption in multivariate Cox models**

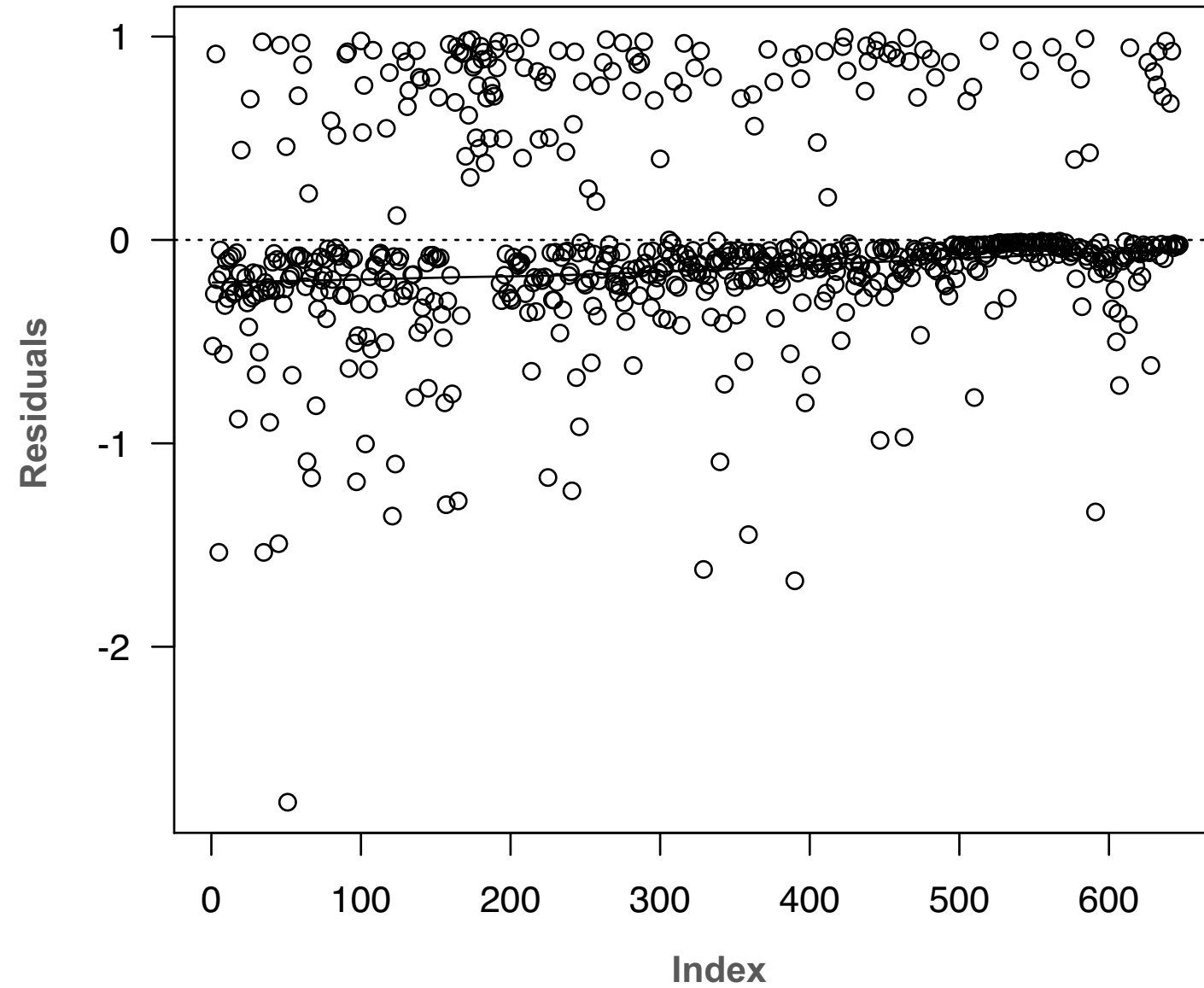

Supplement: Supplementary file 1 — Supplementary Information 1. [file 41598_2024_66463_MOESM1_ESM.pdf]
