# Supplementary material for: Nonlinear association between PD-L1 expression levels and the risk of postoperative recurrence in non-small cell lung cancer
Source: Sci Rep. 2024 Jul 4;14:15369. doi: 10.1038/s41598-024-66463-6 (PMC11224325; doi:10.1038/s41598-024-66463-6)
Supplement: Supplementary file 2 — Supplementary Information 2. [file 41598_2024_66463_MOESM2_ESM.pdf]

Table S1. Hyperparameters for each machine learning model

**Hyperparameters for Random forest**	
n_estimators	8234
max_depth	6463
max_samples	0.579
max_features	0.213
random_state	5301

**Hyperparameters for Gradient boosting**	
n_estimators	191
max_depth	9
learning_rate	0.001
subsample	0.257
max_features	0.416
random_state	7967

**Hyperparameters for Light gradient boosting**	
n_estimators	8975
max_depth	59

learning_rate	0.043
num_leaves	121
min_child_samples	93
subsumple	0.417
colsample_bytree	0.362
random_state	618

**Hyperparameters for Ada boosting**	
n_estimators	71
random_state	967
learning_rate	0.239
