# Supplementary material for: Nonlinear association between PD-L1 expression levels and the risk of postoperative recurrence in non-small cell lung cancer
Source: Sci Rep. 2024 Jul 4;14:15369. doi: 10.1038/s41598-024-66463-6 (PMC11224325; doi:10.1038/s41598-024-66463-6)
Supplement: Supplementary file 3 — Supplementary Information 3. [file 41598_2024_66463_MOESM3_ESM.pdf]

Table S2. Predictive performance of various machine learning models in identifying postoperative lung cancer recurrence in the training cohort

Machine learning model	Assessment metrics (95% CI)				
	ROC AUC	PR AUC	Accuracy	F1 score	Brier score
Random forest	0.99 (0.99–1.00)	1.00 (1.00–1.00)	0.99 (0.97–1.00)	0.98 (0.96–0.99)	0.04 (0.02–0.06)
Gradient boosting	0.97 (0.96–0.99)	0.93 (0.89–0.96)	0.75 (0.70–0.80)	0.67 (0.47–0.86)	0.16 (0.12–0.19)
Light gradient boosting	0.99 (0.98–1.00)	0.98 (0.96–0.99)	0.98 (0.96–0.99)	0.95 (0.93–0.98)	0.03 (0.02–0.05)
Ada boosting	0.86 (0.82–0.91)	0.67 (0.62–0.73)	0.82 (0.77–0.86)	0.54 (0.49–0.61)	0.16 (0.13–0.19)

Abbreviations: CI, confidence interval; PR AUC, area under the precision-recall curve; ROC AUC, area under the receiver operating characteristic curve.
