# Supplementary material for: Nonlinear association between PD-L1 expression levels and the risk of postoperative recurrence in non-small cell lung cancer
Source: Sci Rep. 2024 Jul 4;14:15369. doi: 10.1038/s41598-024-66463-6 (PMC11224325; doi:10.1038/s41598-024-66463-6)
Supplement: Supplementary file 4 — Supplementary Information 4. [file 41598_2024_66463_MOESM4_ESM.pdf]

Table S3. Results of a multivariate Cox proportional hazard analysis of RFS according to the expression of PD-L1.

	**Unadjusted HR (95% CI), *P***	**[†]Adjusted HR [a] (95% CI), *P***	**VIF**
**Continuous variable**			
PD-L1 expression (TPS [%])	1.013 (1.008–1.018), <0.001	1.006 (1.000–1.011), 0.04	1.09
NLR	1.08 (1.01–1.15), 0.02	1.08 (1.01–1.17), 0.03	1.01
**Categorical variables**			
Age (<65)	Reference	Reference	1.05
Age (≥65)	0.81 (0.58–1.15), 0.25	0.85 (0.58–1.24), 0.39	
Sex			1.06
Men	Reference	Reference	
Women	0.50 (0.35–0.72), <0.001	0.72 (0.49–1.05), 0.08	
Histological type			1.07
Adenocarcinoma	Reference	Reference	
Squamous cell carcinoma	1.74 (1.18–2.56), 0.005	1.25 (0.81–1.93), 0.32	
Others[‡]	2.58 (1.62–4.11), <0.001	1.32 (0.79–2.19), 0.29	
Pathological Stage			1.08

I	Reference	Reference	
II	3.50 (2.31–5.28), <0.001	2.29 (1.48–3.55), <0.001	
III	8.48 (5.73–12.56), <0.001	6.70 (4.32–10.38), <0.001	
Vascular invasion			1.18
v0	Reference	Reference	
v1	4.82 (2.73–8.52), <0.001	2.53 (1.27–5.05), <0.001	
Lympho-vessel invasion			1.17
Ly0	Reference	Reference	
Ly1	2.56 (1.69–3.89), <0.001	0.87 (0.52–1.44), 0.57	
Adjuvant chemotherapy			1.15
No adjuvant therapy	Reference	Reference	
Platinum-based chemotherapy	2.59 (1.76–3.82), <0.001	0.71 (0.44–1.12), 0.14	

[†]Adjusted for age, sex, histological type, pathological stage, vascular invasion, lympho-vessel invasion and adjuvant chemotherapy.

[‡]Defined as histological types of NSCLC with the exclusion of AD and SCC. Among the 53 patients, 22 had pleomorphic carcinoma, 13 had large-cell neuroendocrine carcinoma, 11 had adenosquamous carcinoma and 7 had large-cell carcinoma.

Abbreviations: *RFS* recurrence-free survival*, PD-L1* programmed death-ligand 1, *NLR* neutrophil-to-lymphocyte ratio, *HR* hazard ratio, *CI* confidence interval, *VIF* variance inflation factor
