# Supplementary material for: Nonlinear association between PD-L1 expression levels and the risk of postoperative recurrence in non-small cell lung cancer
Source: Sci Rep. 2024 Jul 4;14:15369. doi: 10.1038/s41598-024-66463-6 (PMC11224325; doi:10.1038/s41598-024-66463-6)
Supplement: Supplementary file 5 — Supplementary Information 5. [file 41598_2024_66463_MOESM5_ESM.docx]

Table S4. Testing the proportional hazards assumption in Cox models for RFS.

| Explanatory variables in the Cox models | *P*^‡^ |
| --- | --- |
| PD-L1 expression (TPS [%]) | 0.36 |
| NLR | 0.94 |
| Age (≥65) (reference: <65) | 0.22 |
| Sex: female (reference: male) | 0.68 |
| Squamous cell carcinoma (reference: adenocarcinoma)  Others^†^ (reference: adenocarcinoma) | 0.42  0.20 |
| Pathological stage II (reference: pathological stage I) | 0.16 |
| Pathological stage III (reference: pathological stage I) | 0.26 |
| Vascular invasion (v1) (reference: v0) | 0.59 |
| Lymph-vessel invasion (L1) (reference: L0) | 0.77 |
| Platinum-based chemotherapy (reference: No adjuvant therapy) | 0.39 |

^†^Defined as histological types of NSCLC with the exclusion of AD and SCC. Among the 53 patients, 22 had pleomorphic carcinoma, 13 had large-cell neuroendocrine carcinoma, 11 had adenosquamous carcinoma and 7 had large-cell carcinoma.

^‡^Schoenfeld residual test

*Abbreviations*: *RFS* recurrence-free survival*, PD-L1* programmed death-ligand 1, *NLR* neutrophil-to-lymphocyte ratio
